# Clinical Diagnoses Made Before and After Implementing Best Practices to Improve Diagnostic Quality in a Psychogeriatric Clinic in Sri Lanka

**DOI:** 10.1192/bjo.2026.11433

**Published:** 2026-06-30

**Authors:** Amodha Medagedara, MGN Priyangani, DVM Dias, Kapila Ranasinghe

**Affiliations:** 1 East London NHS Foundation Trust, Luton, United Kingdom; 2 Lecturer in Pharmacology, Faculty of Medicine, University of Kelaniya, Ragama, Sri Lanka; 3 NIMH, Angoda, Sri Lanka

## Abstract

**Aims:**

Deeghayu clinic at the National Institute of Mental Health, Sri Lanka is in the forefront of serving psychogeriatric disorders which are in the rise. A project was launched in the latter half of 2023 to improve quality of diagnosis made there. It included implementation of best practices such as limiting number of patients seen per day while increasing days of contact to increase time spent on each and number viewed per week, usage of Montreal Cognitive Functional Assessment scale(MoCA) and Bristol scale in all the patients and other cognitive, functional and disease-specific assessments only as required and presenting a multidisciplinary assessment to the Consultant Psychiatrist to arrive at a diagnosis. Thus the aim of this study is to assess improvement in diagnostic precision made after implementation of best practices.

**Methods:**

Two audits were done comparing clinical diagnosis made before (first six months of 2023) and after implementing best practices (first 6 months of 2024).

**Results:**

Total number of patients viewed had soared from 43 to 95. Proportion of patients who had undergone MoCA and Bristol scale had soared from 86% and 39.5% respectively to 104.2% (including re-assessments to ascertain response to therapy) and 95.7%. Variety of diagnosis made had risen from identifying 22, 1, 1, 5 patients with Alzheimer’s, vascular, Lewy body and mixed dementia to identifying 10, 14, 5 patients with mild, moderate and severe Alzheimer’s dementia respectively and 10, 7, 1 patients with mild, moderate andsevere vascular dementia, 5 other varying types of dementia and 5 with mild cognitive impairment. Identifying 14 with depression in 2023 had risen to 11, 11 and 3 with mild, moderate and severe depression.

**Conclusion:**

Multidisciplinary approach, provision of adequate time per each patient and doing essential assessments in all patients and others as required to supplement the diagnosis can improve the precision and quality of diagnosis made despite a lack of sophisticated facilities and infrastructure. Thus this represents a scalable model that can be replicated across other low-income country settings as well.

